# Quantitative Study of Individual Creative Caregiving Sessions on Well-Being of Caregivers and Care Recipients

**DOI:** 10.1093/geroni/igaa057.244

**Published:** 2020-12-16

**Authors:** Felicia Wheaton, Safiyyah Cole, Sai Raj Kappari, Matilda Johnson

**Affiliations:** 1 Bethune-Cookman University, Daytona Beach, Florida, United States; 2 Westcliff University, Daytona Beach, Florida, United States; 3 Bethune-Cookman University, Bethune-Cookman University, Florida, United States

## Abstract

There were approximately 34.2 million unpaid caregivers of adults age 50+ in the United States in the last 12 months (NAC & AARP, 2015). These individuals provide important care for older adults with physical, psychological and cognitive problems. There is a growing awareness that caregivers are also at risk for physical and mental health problems and therefore also require support to reduce stress and maintain optimal health. Research suggests that engaging in creative and artistic activities may reduce stress and improve physical and mental health among caregivers. Researchers from Bethune-Cookman University partnered with the Atlantic Center for the Arts in New Smyrna Beach, FL to evaluate their Creative Caregiving program. Approximately 10 informal caregivers and their care partners met for 2 hours each week over the course of 6 weeks from February 3-March 9, 2020. Participants learned how to connect mind, body and spirit by using the arts as a tool of self-care, social interaction, and learning. At each session, participants were asked to rate their overall wellbeing on a scale from 1-10. Pre- and post-session data was analyzed using paired samples t-tests. Results indicate that there was significant improvement in wellbeing and participants reported improvements in their stress, mood and relationship with their caregiver or care partner. These findings were observed for both caregivers and care partners, suggesting that arts programs can benefit both. Such programs are a fun and cost-effective way to improve wellbeing, at least in the short-term.

